# Assessment of CRISPRa-mediated *gdnf* overexpression in an *In vitro* Parkinson’s disease model

**DOI:** 10.3389/fbioe.2024.1420183

**Published:** 2024-08-08

**Authors:** Paula Guzmán-Sastoque, Sebastián Sotelo, Natalia P. Esmeral, Sonia Luz Albarracín, Jhon-Jairo Sutachan, Luis H. Reyes, Carolina Muñoz-Camargo, Juan C. Cruz, Natasha I. Bloch

**Affiliations:** ^1^ Biomedical Engineering Department, Universidad de los Andes, Bogotá, Colombia; ^2^ Departamento de Nutrición y Bioquímica, Pontificia Universidad Javeriana, Bogotá, Colombia; ^3^ Department of Chemical and Food Engineering, Grupo de Diseño de Productos y Procesos (GDPP), Universidad de los Andes, Bogotá, Colombia

**Keywords:** CRISPR gene overexpression, Parkinson’s disease model, GDNF, nanoparticle delivery system, oxidative stress

## Abstract

**Introduction:**

Parkinson’s disease (PD) presents a significant challenge in medical science, as current treatments are limited to symptom management and often carry significant side effects. Our study introduces an innovative approach to evaluate the effects of *gdnf* overexpression mediated by CRISPRa in an *in vitro* model of Parkinson’s disease. The expression of *gdnf* can have neuroprotective effects, being related to the modulation of neuroinflammation and pathways associated with cell survival, differentiation, and growth.

**Methods:**

We have developed a targeted delivery system using a magnetite nanostructured vehicle for the efficient transport of genetic material. This system has resulted in a substantial increase, up to 200-fold) in *gdnf* expression in an *In vitro* model of Parkinson’s disease using a mixed primary culture of astrocytes, neurons, and microglia.

**Results and Discussion:**

The delivery system exhibits significant endosomal escape of more than 56%, crucial for the effective delivery and activation of the genetic material within cells. The increased *gdnf* expression correlates with a notable reduction in MAO-B complex activity, reaching basal values of 14.8 μU/μg of protein, and a reduction in reactive oxygen species. Additionally, there is up to a 34.6% increase in cell viability in an *In vitro* Parkinson’s disease model treated with the neurotoxin MPTP. Our study shows that increasing *gdnf* expression can remediate some of the cellular symptoms associated with Parkinson’s disease in an *in vitro* model of the disease using a novel nanostructured delivery system.

## 1 Introduction

One of the primary etiological factors contributing to Parkinson’s disease (PD) is the progressive degeneration and subsequent demise of dopaminergic neurons situated within the Substantia Nigra pars compacta (SNpc) region of the brain (Emamzadeh and Surguchov, 2018; Bloem et al., 2021; Faial, 2024; Mahato and Saarma, 2024). Following Alzheimer’s disease, Parkinson’s disease stands as one of the most frequent neurodegenerative disorders worldwide (Jankovic, 2008; Troncoso-Escudero et al., 2018), affecting over 8.5 million individuals globally by 2019 (Nakmode et al., 2023). Manifestations of motor dysfunction characteristic of PD encompass bradykinesia, resting tremors, rigidity, and postural instability. Non-motor symptoms encompass psychosis, depression, anxiety, pain, and fatigue (Sivanandy et al., 2021). Consequently, PD significantly impacts the quality of life for afflicted individuals (Al-Khammash et al., 2023). Currently, Parkinson’s disease (PD) lacks a definitive cure (Pardo-Moreno et al., 2023), and the primary therapeutic approach involves pharmacological interventions aimed at managing symptoms, underscoring an urgent need for innovative new and curative therapeutic strategies. The existing pharmacopeia for PD—spanning from the mainstay levodopa to a suite of dopamine agonists, COMT inhibitors, anticholinergics, MAO-B inhibitors, amantadine, safinamide, istradefylline, and pimavanserin—offers symptomatic respite but falls short of halting disease progression (Armstrong and Okun, 2020; Jankovic and Tan, 2020).

The advent of CRISPR-Cas technology has opened-up the use of gene editing and gene expression manipulation as standard research tools, and even possible avenues for treatment development due to their efficiency, cost-effectiveness, relative safety, and ease of use (Tavakoli et al., 2021). CRISPR-Cas constitutes a microbial adaptive immune system employing RNA-guided nucleases to cleave foreign genetic elements (Ran et al., 2013). The Type II CRISPR system comprises the nuclease Cas9, the crRNA array encoding guide RNAs, and an essential auxiliary trans-activating crRNA (tracrRNA), facilitating crRNA processing (Tang and Fu, 2018). Among CRISPR-Cas tools, CRISPR/sgRNA-directed synergistic activation mediator (SAM), or CRISPRa, offers the possibility to activate gene expression and, thus, overexpress a target gene in a controlled manner (Sokka et al., 2022). CRISPRa is characterized by the deactivation of the Cas9 enzyme, rendering it devoid of endonuclease activity (dCas9) (Pinjala et al., 2023). This toolkit presents a unique opportunity to investigate the potential of gene expression manipulation by enabling the overexpression or activation of endogenous genes implicated in the pathways that become altered in PD (Giehrl-Schwab et al., 2022; Narváez-Pérez et al., 2024). CRISPRa has multiple advantages, mainly its efficiency, cost-effectiveness, low off-target rate, independence of the target gene size and mutations, (Becirovic, 2022). CRISPRa has recently been proposed in the literature as a therapeutic option for Parkinson’s disease (Pinjala et al., 2023; Sahel et al., 2023).

Notwithstanding, the challenges inherent to the delivery of CRISPR elements to the target cells remain a formidable barrier (van Haasteren et al., 2020). Traditional delivery methods can trigger hyperactive immune responses (Freitas et al., 2022), lysosomal degradation, and unwanted impacts at sites that are not of interest (off-targets) (Lee and Ahn, 2018; Charlesworth et al., 2019). The use of non-viral delivery vehicles (Wang et al., 2024b) is a promising technology for genetic material delivery (Mendes et al., 2022). Nano and micro particles have garnered significant interest within the scientific community due to their multifaceted potential applications (Safarik and Safarikova, 2009; Hamilton et al., 2023; Rodríguez et al., 2023; Rodríguez et al., 2024). For instance, nanostructured vehicles have been used as an efficient method for delivering genetic material into the cells (Sachdeva et al., 2022; Unnisa et al., 2023). Magnetite nanoparticles (MNPs) have high magnetic susceptibility, high biocompatibility, low production costs, facile functionalization, and homogeneous morphology (Sachdeva et al., 2022). MNPs have garnered attention in biomedical and biotechnological applications (Rodriguez et al., 2021; Dash et al., 2022; Tran et al., 2022). To enhance their capability for genetic material delivery, MNPs can be functionalized with membrane translocating peptides, such as Buforin II (BUF-II), which has demonstrated efficacy in delivering nucleic acids into cells (Cuellar et al., 2018; Arango et al., 2023). MNPs can thus be an excellent delivery vehicle for gene editing elements with enormous potential in research and the design of future gene therapies.

Among the pathways and cellular components disrupted during Parkinson’s disease (PD), mitochondrial dysfunction plays a significant role in the progression of the disease (Henrich et al., 2023). In this context, glial cell line-derived neurotrophic factor (GDNF) emerges as a crucial factor in preserving mitochondrial structure and function by activating the NF-κB transcription factor. This activation is mediated by RET kinase through the phosphoinositide-3-kinase (PI3K) pathway (Mitroshina et al., 2019; Shishkina et al., 2018). Henrich et al. (2023). GDNF is involved in neuronal growth and survival, and it is integral to the MAP kinase and PI3 kinase pathways—both of which are instrumental in the maintenance and functionality of dopaminergic neurons in the midbrain (Mol et al., 2023). Specifically, GDNF first forms a complex with GFRα1 and then binds to the RET receptor tyrosine kinase, leading to the activation of signaling cascades and enzymatic pathways related to cell survival, differentiation, and growth (Kawai and Takahashi, 2020; Kotliarova and Sidorova, 2021). In PD, GDNF’s association with neuronal resilience to oxidative stress—a known contributor to dopaminergic neuron demise—renders it a prime candidate for overexpression (Merola et al., 2020; Miyazaki and Asanuma, 2020; Bloem et al., 2021; Sreepad et al., 2021). The potential of GDNF as a regenerative and neuroprotective agent has been corroborated in several preclinical and clinical settings, positioning it as an attractive target for gene therapy in PD (Barker et al., 2020; Duarte Azevedo et al., 2020; Manfredsson et al., 2020; Xiao et al., 2022).

Here, our objective is to evaluate the effects of *gdnf* overexpression mediated by CRISPRa in an *in vitro* model of Parkinson’s disease. Leveraging its biocompatibility and chemical versatility, we employed a magnetite nanostructured delivery system within a well-established *in vitro* Parkinson’s disease model to systematically investigate the cellular impact of *gdnf* gene overexpression. This was achieved using a co-culture of neurons, astrocytes, and microglia treated with the neurotoxin MPTP (1-methyl-4-phenyl-1,2,3,6-tetrahydropyridine hydrochloride) (Zhang et al., 2017). We meticulously standardized the delivery protocol for the CRISPRa components using functionalized magnetite nanoparticles, ensuring maximum efficiency in genetic material delivery. To comprehensively evaluate the effects of *gdnf* overexpression, we analyzed cellular responses and the resulting alterations in the *in vitro* PD model, focusing on cell viability, gene expression profiles, reactive oxygen species levels, and MAO-B activity. Our hypothesis posits that CRISPRa, combined with a magnetite nanobioconjugate delivery system, will significantly enhance *gdnf* expression, mitigate oxidative stress, and promote cellular proliferation, thereby alleviating the detrimental cellular environment induced by MPTP. This innovative approach aims to uncover additional benefits of *gdnf* expression.

## 2 Materials and methods

Some protocols followed are described in more detail in a previous article (Arango et al., 2023) elaborated by researchers from the Department of Biomedical and Chemical Engineering at Universidad de los Andes.

### 2.1 Materials

Cellular Reactive Oxygen Species Detection Assay Kit (Deep Red Fluorescence) (ab186029), Astrocyte Marker (ALDH1L1, EAAT1, EAAT2, GFAP) Antibody Sampler Panel (ab226481) and Monoamine Oxidase (MAO) Assay Kit (ab241031) were purchased from Abcam (Cambridge, United Kingdom). Dimethyl sulfoxide (99.5%), tetramethylammonium hydroxide (TMAH) (25%), (3-aminopropyl) triethoxysilane (APTES) (98%), N-[3-(dimethylamino propyl]-N’-ethyl carbodiimide (EDC) (98%), glutaraldehyde (25%), NH2-PEG12-Propionic acid, Triton X-100, MPTP (1-methyl-4-phenyl-1,2,3,6-tetrahydropyridine hydrochloride) and LB Broth Lennox, were purchased from Sigma-Aldrich (St. Louis, MO, United States). Iron (III) chloride 6-hydrate pure, acetic acid (99.5%), and sodium hydroxide (NaOH) (98%) were obtained from Panreac AppliChem (Barcelona, Spain). Iron (II) chloride tetrahydrate (98%) was purchased from Alfa Aesar (Haverhill, MA, United States). 3-[(2-Aminoethyl) dithio] propionic acid hydrochloride (AEDP) was purchased from Chem Impex (Wood Dale, IL, United States). Dulbecco’s Modified Eagle Medium (DMEM) and Fetal Bovine Serum (FBS) were purchased from BioWest (Riverside, MO, United States). Buforin II (BUF-II-TRSSRAGLQFPVGRVHRLLRK) was synthesized by GL Biochem Shanghai (Shanghai, China).

Primers, sgRNA, thiolated tag, EcoRI-HF Enzyme (NEB-R3101S), PvuI-HF Enzyme (NEB-R3150S), Q5 High-Fidelity 2X Mastermix (NEB-M0515), Monarch PCR & DNA Cleanup Kit (NEB-T1030S), and Monarch Plasmid Miniprep kit (NEB-T1010S) were obtained from New England Biolabs (Ipswich, MA, United States). pCas-guide-CRISPRa (#GE100055) and pCas-Enhancer (#GE100056) were obtained from Origene (Rockville, MD, United States). The isolation of the primary culture of rat astrocytes, microglia, and neurons will be described in the methodology section 2.9.

### 2.2 MNP synthesis

The synthesis of the magnetic nanoparticles (MNPs) was performed by co-precipitation of iron (III) chloride and iron (II) chloride in Type I water (resistivity >1 MΩ-cm and conductivity <1 μS/cm) at 0°C and a 2:1°M ratio, precisely as described in previous articles (Cifuentes et al., 2023). Subsequently, the obtained solution was transferred to a double-detachment flask, and 1 M NaOH solution was added dropwise to the solution under nitrogen flow (0.5 L/min) and mechanical stirring at 200 rpm for 1 hour.

Finally, three washes with NaCl (1.5% *w/v*) and two with Type I water were performed to remove excess reagents. The MNPs were magnetically precipitated with neodymium magnets and resuspended using a Branson 2,800 Series ultrasonic cleaner.

### 2.3 Silanization and surface functionalization of MNPs

The MNPs underwent silanization using APTES, which was carefully chosen based on its demonstrated efficacy in previous studies for facilitating subsequent bioconjugation (Ortegón et al., 2022; Arango et al., 2023; Cifuentes et al., 2023). Briefly, 100 mg of MNPs were resuspended in 40 mL of type I water by sonicating for 5 min using a Branson 2,800 Series ultrasonic cleaner (Danbury, CT, United States). Subsequently, 250 μL of TMAH, 50 μL of acetic acid, and 1 mL of APTES (20% v/v) were added to the reaction mixture. Following the sequential addition of each chemical reagent, the suspension of MNPs was subjected to ultrasonic dispersion for 1 min and then agitated for 3 min to ensure thorough homogenization of the mixture. The combined MNPs and reagents were then stirred continuously at 220 rpm and a temperature of 60°C for 1 hour. The suspension was washed three times with a 1.5% (w/v) NaCl solution, followed by two rinses with Type I water to remove unreacted reagents and prevent nanoparticle aggregation.

To further enhance the stability of the MNPs and avoid aggregation (Suk et al., 2016), the MNPs underwent PEGylation with NH2-PEG12-Propionic acid (PEG) with glutaraldehyde as the crosslinker agent. For this, 2 mL of glutaraldehyde 2% (v/v) was added to each 100 mg of Organosilane-MNPs (Si-MNPs) resuspended in Type I water. The suspension was then stirred at 180 rpm for 1 h. Then, PEG was added at a 3:1 M ratio to the amount of amine groups present on the surface of the MNPs, and the reaction was left under mechanical stirring overnight to promote conjugation. The PEGylated MNPs were washed with NaCl 1.5% (w/v) and Type I water to remove excess reagents.

Subsequently, MNPs were further functionalized with 3-[(2-Aminoethyl) dithio] propionic acid hydrochloride (AEDP) to introduce disulfide bonds that facilitate the conjugation of thiolated DNA (tDNA) via a disulfide exchange reaction, as detailed in existing literature (Yi and Khosla, 2016; Altinbasak et al., 2020). The carboxyl groups of the PEGylated MNPs were activated with 30 mg of EDC and 15 mg of NHS in aqueous suspension, then adding 5 mg of AEDP. The solution was then incubated at 38°C for 10 min to ensure activation and then stirred at 220 rpm overnight, promoting the formation of AEDP-PEG-MNP nanoconjugates. Subsequent washes with NaCl solution and Type I water removed any unreacted reagents.

For the conjugation of tDNA, 10 mg of AEDP-PEG-MNP nanoconjugates were resuspended in 5 mL of Type I water, combined with 5 mL of 20 mM DTT, and 25 µg of tDNA, in preparation for disulfide exchange as illustrated in Supplementary Figure S1. The mixture was placed in a dialysis cassette equipped with a 3.5 kDa MWCO membrane and dialyzed against 800 mL of Type I water for 24 h, with water changes every 2 hours. The process reduced the disulfide bonds in AEDP, forming terminal thiol groups that react with tDNA under controlled oxidative conditions to reform a disulfide bond (Tsuruoka et al., 2020).

Finally, the BUF-II translocating peptide was conjugated to the tDNA-AEDP-PEG-MNP complex to facilitate cellular uptake (Perez et al., 2019). In this final step, 10 mg of tDNA-AEDP-PEG-MNP were resuspended in 15 mL of Type I water, and a mixture of 3 mg of EDC (crosslinking agent) and 1.5 mg of NHS was added to activate the carboxyl groups of PEG. The solution was then incubated at 38°C for 10 min before adding BUF-II in a 3:1 M ratio to the tDNA-AEDP-PEG-MNP solution. The final conjugates were thoroughly washed with NaCl solution and Type I water to purify the product.

### 2.4 Physicochemical characterizations

To validate each functionalization step of the MNPs, we employed a suite of physicochemical characterization techniques. Hydrodynamic diameter measurements were performed using dynamic light scattering (DLS) using a Zeta-Sizer Nano ZS instrument (Malvern, United Kingdom), confirming an anticipated increase in the size of the nanoconjugates post-functionalization. Surface charge assessment, crucial for predicting nanoparticle behavior in biological environments, was determined through zeta potential analysis using the same instrument. Fourier-transform infrared (FTIR) spectroscopy, conducted with a Thermo Scientific Nicolet iS50 FTIR spectrometer (Waltham, MA, United States), provided insight into successfully incorporating functional groups onto the MNP surface. Each spectrum was analyzed to confirm the presence of characteristic peaks corresponding to the functional groups introduced at each stage of functionalization. To further assess our nanoconjugates’ structural integrity and thermal stability, thermogravimetric analysis (TGA) was conducted using a TA Instruments TGA Q50 (New Castle, DE, United States). This analysis measured the weight loss of the MNPs from 30°C to 800°C at a heating rate of 10°C/min under a nitrogen atmosphere, revealing the thermal decomposition profile and confirming the stability of the functional groups up to the relevant physiological temperatures.

These physicochemical characterizations are indispensable for determining the nanobioconjugate’s suitability as a delivery system. The particle size influences cellular internalization, while surface charge affects dispersion stability, cellular interaction, and the bio-distribution of the nanoparticles (Mazumdar et al., 2021). Thermal stability is also a critical parameter, particularly for the potential of these nanoconjugates to withstand physiological conditions without premature degradation or release of the therapeutic payload.

### 2.5 Synthesis and conjugation of CRISPRa elements

The sgRNA constructs were engineered to upregulate *gdnf* expression, adhering to methods detailed by previous studies (Merola et al., 2020). The sgRNA sequences were selected using the GPP sgRNA Designer tool developed by the Broad Institute (Doench et al., 2016). The selection criteria prioritized guides that minimized off-target effects and maximized activity at the desired genomic locus. The specific sgRNA sequences and the criteria for their selection are comprehensively detailed in Supplementary Table S1. The efficiency of the guide RNAs was validated using RT-qPCR, with results illustrated in Supplementary Figure S4. Lipofectamine 3,000^®^ and the accompanying P3000 enhancer were employed for cell transfection purposes.

sgRNA was inserted into the CRISPRa plasmid with VP64 activation domain (8.2 Kb plasmid, “CRISPRa—CRISPR/Cas9 SAM Synergistic Activation Mediator"–Origene GE100057) (Supplementary Figure S5; Supplementary Figure S6). Plasmid was cloned on Escherichia coli DH10B competent cells, with the plasmid presence confirmed via standard molecular biology techniques. Furthermore, we cloned the CRISPRa-Enhancer vector (6.3 Kb plasmid), which contains the p65 and HSF1 activation domains, to further potentiate gene activation (Supplementary Figure S7). To prepare these CRISPRa components for MNP conjugation, the sgRNA and scaffold complex were linearized and amplified using a thiol-modified reverse primer (Supplementary Table S2). The strategic introduction of a thiol group at the 3’ of the DNA elements enables covalent attachment to the nanoparticles, forming the AEDP-PEG-MNP nanoconjugates. These DNA-MNP conjugates, linked by a disulfide bond (Tsuruoka et al., 2020), are primed to release the CRISPRa elements within the reducing environment of the cytoplasm. All amplifications were carried out with a high-fidelity polymerase (Q5 High-Fidelity MasterMix 2X) and the Eppendorf^®^ Mastercycler^®^ Nexus Thermal Cycler (Waltham, MA, United States) to prevent the risk of mutations in the CRISPR elements. Finally, linearized DNA constructs were purified (Monarch DNA and PCR cleanup kit), and their integrity was validated by agarose gel electrophoresis (Supplementary Figure S8).

### 2.6 Validation of the conjugation of the CRISPRa system to the nanoparticle

To confirm the successful attachment of CRISPRa components to the nanoparticle delivery system, we employed a validation protocol reported previously (Arango et al., 2023). The CRISPRa system (tDNA) was fluorescently labeled using 10X GelRed^®^ (Sapia et al., 2021) for visualization, as shown in Supplementary Figure S2. This labeled tDNA was then conjugated to the AEDP-functionalized nanoparticles (AEDP-MNP). Post-conjugation, the nanoparticles underwent washing with NaCl and Type I water, as previously described.

The delivery of the GelRed®-labeled nanobioconjugate (*gdnf* (++)-MNP-BUF-II), from henceforth on referred to as *gdnf-*CRISPRa nanobioconjugate, was performed in a mixed co-culture system composed of astrocytes, neurons, and microglia. For this, 70,000 cells were seeded per well in a 12-well microplate containing glass coverslips pre-treated with poly-d-lysine at 50 μg/mL, with DMEM medium supplemented with 10% FBS and 1% penicillin/streptomycin (P/S). After delivering the labeled nanobioconjugate to cells and 0.5 h incubation, we performed a wash with 1X PBS to remove non-internalized nanoparticles. This was followed by the co-delivery of the nuclear stain Hoechst (1:1,000) and the endosomal marker LysoTracker Green^®^ DND-26 (1:10,000) to facilitate the visualization of cell nuclei and endosomes, respectively. Confocal microscopy was performed using an Olympus FV1000 microscope with a 40X objective to visualize and confirm nanoparticle uptake and intracellular localization. The nuclei, lysosomes, and nanoparticles were identified using excitation/emission wavelengths 405/461 nm, 488/535 nm, and 559/600 nm, respectively. Fiji^®^ software was used for image analysis (Supplementary Figure S9).

### 2.7 Isolation of rat cells (mixed primary cell culture)

Primary cultures were derived from neonatal Wistar rats (males of *Rattus norvergicus*, postnatal days 3–5, n = 4) held at the Pontificia Universidad Javeriana, Bogotá, Colombia. All our protocols for animal handling and euthanasia followed those reported previously (Arango et al., 2023) and were approved by the Committee for Animal Care and Use at Pontificia Universidad Javeriana (Minute Number FUA 144-22). The animals were euthanized by decapitation to avoid stress, consistent with the university’s animal welfare guidelines. Following the protocol established in the FUA, then, heads were disinfected with 70% (v/v) ethanol before skin and skull removal under a ZEISS Stereo Discovery.V12 stereomicroscope (Oberkochen, Germany). Brain extraction was performed with care, and the tissues were immediately placed in Hanks Balanced Salt Solution (HBSS) buffer containing 1% P/S and phenol red to maintain cellular viability. Under the stereoscope, we meticulously separated the cerebral cortices, ensuring the removal of the olfactory bulb, cerebellum, meninges, and any vascular tissue. The tissues underwent enzymatic disintegration with 10X Trypsin for 20 min at 37 C and 5% CO_2_, followed by mechanical disaggregation by resuspension with micropipettes and tips of different volumes (1,000 μL, 100 μL, and 10 µL). The resultant cell suspension was filtered through a 40-µm mesh to ensure a uniform single-cell solution. The isolated cells were then resuspended in 10 mL of DMEM supplemented with 10% FBS and 1% P/S, seeded at a density of 300,000 cells/mL, which is conducive to simulating the neural microenvironment necessary for our experimental objectives. The experiments were carried out 8 days after cell isolation. The resulting cultures comprised astrocytes, responsible for metabolic, water and ion homeostasis critical to neuronal function; microglia, the resident immune cells of the central nervous system (Jäkel and Dimou, 2017); neurons, the principal signaling units of the brain (Akay, 2023); and oligodendrocytes, thus providing a representative model of the central nervous system for evaluating nanobioconjugate delivery (Supplementary Figure S10).

To quantify the proportion of astrocytes, neurons, and microglia in the extracted culture, we performed a staining procedure using GFAP, EAAT1, and EAAT2 antibodies (ab226481). A total of 20,000 cells per well were seeded in a 12-well microplate. First, the cells were washed with 1X PBS, followed by the addition of 50 µL of 4% formaldehyde and incubation for 10 min. Subsequently, 500 µL of 0.3% Triton X-100 was added, and cells were incubated for 5 min. After membrane permeabilization, 500 µL of 3% albumin was added and incubated for 1 h. Finally, we incubated with the primary antibody (anti-GFAP antibody or anti-EAAT1 antibody or anti-EEAT2 antibody) at a 1:5,000 dilution overnight at 4°C, followed by the addition of the anti-Rabbit IgG (HRP) secondary antibody at a 1:1,000 dilution (Supplementary Figure S11). We captured images using confocal microscopy (Olympus FV1000 microscope), with a 40X objective. The excitation/emission wavelength (nm) for GFAP, EAAT1, and EAAT2 was set at 488/535 nm.

### 2.8 Endosomal escape

To evaluate the endosomal escape of the *gdnf-*CRISPRa nanobioconjugate, the nanoconjugate was labeled with Rhodamine-B, as shown in Supplementary Figure S3. In preparation for conjugation, 15 mg of NHS and 30 mg of EDC were dissolved in 5 mL of Type I water and added to 100 mg of the nanobioconjugate. To this reactive mix, 5 mg of Rhodamine-B and 2 mL of a 50% (v/v) DMF were added. The reaction mixture was then heated to 38°C and magnetically stirred for 10 min, a step crucial for activating Rhodamine-B’s carboxyl groups. Subsequently, the Rhodamine-B solution was combined with the nanobioconjugates and stirred at 180 rpm for 24 h to ensure thorough conjugation. Excess reagents were removed through a series of washes with NaCl and Type I water, as previously described.

The delivery of the previously labeled nanobioconjugate (*gdnf* (++)-MNP-BUF-II-RhodB), from henceforth on referred to as Rhodamine-B-labeled nanobioconjugate, was carried out in a mixed cell culture consisting of astrocytes, neurons, and microglia. The culture protocol involved seeding 70,000 cells per well in a 12-well microplate, with each well-containing glass coverslips pre-coated with poly-d-lysine at 50 μg/mL. The medium for cell culture was DMEM supplemented with FBS and P/S. The cells and labeled nanobioconjugate were incubated at 37°C with 5% CO_2_ and 95% humidity.

After incubating for 0.5, 4, or 7 h at 37°C with the Rhodamine-B labeled nanobioconjugate, cultures were washed with 1X PBS. This was followed by the co-delivery of the Hoechst nuclear stain (1:1,000) and the LysoTracker Green^®^ DND-26 endosomal marker (1:10,000). Imaging was conducted using confocal microscopy with an Olympus FV1000 microscope, utilizing a 40X objective. The excitation/emission wavelengths (nm) for nuclei, lysosomes, and nanoparticles were set at 405/461, 488/535, and 559/600, respectively. For each time point, ten images were captured, and a minimum of 10 cells were analyzed per image. Fiji^®^ software facilitated the image analysis, providing quantitative data on nanoparticle internalization and endosomal escape.

### 2.9 *In vitro* Parkinson’s disease model

To evaluate the effects of overexpressing *gdnf*, it was necessary to standardize an *in vitro* Parkinson’s disease model using MPTP (Ma et al., 2023; Omar et al., 2023; Ruan et al., 2023). Upon metabolism, MPTP is converted to its oxidized product, 1-methyl-4-phenylpyridinium (MPP+), which is known to induce morphological and functional changes in cells similar to those observed in PD, as per recent findings (Dovonou et al., 2023). We conducted a cell viability assay using lactate dehydrogenase (LDH) to determine the neurotoxin concentration that would cause minimal and significant cellular changes, testing a range from 500 μM to 1.5 mM. We included a positive control with cells exposed to 50 µL of 10% v/v Triton X-100 and a negative control of cells cultured in DMEM. The cell viability percentage (CV%) was calculated according to Eq. 1:

$$CV\%=\left(1-\frac{OD_{test}-OD_{negative}}{OD_{positive}-OD_{negative}}\right)*100$$ (1)

### 2.10 *gndf* RT-qPCR quantification

For RT-qPCR quantification of *gndf*, cells were seeded at a density of 70,000 cells per well in a 24-well microplate with DMEM supplemented with 10% FBS. After 24 h of incubation at 37°C and 5% CO_2_, the medium was replaced with DMEM containing MPTP to induce PD-like cellular changes. Cells were incubated with the neurotoxin (MPTP) at 37 °C in 5% CO_2_ for 24 h. Control cells were maintained in an MPTP-free medium.

Post MPTP exposure for 24 h, we washed the cells twice with 1X PBS and introduced the CRISPRa system by adding DMEM mixed with 50 µg of (CRISPRa construct)-*gdnf*-CRISPRa nanobioconjugate and 15 µg of (CRISPRa enhancer)-CRISPRa nanobioconjugate. Control groups included cells treated with 65 µg of AEDP-PEG-MNP-BUF-II without MPTP exposure, cells exposed to 500 µM MPTP and 1.25 mM MPTP without the nanoconjugate, cells with CRISPRa nanobioconjugate and without exposure to MPTP and cells transfected with lipofectamine 3,000^®^+ P3000 reagent instead of the nanoconjugate and with exposure to MPTP at 500 µM and MPTP at 1.25 mM.

RNA extraction from cells was then performed with the Luna^®^ Cell Ready Lysis Module kit, following the manufacturer’s instructions. RT-qPCR was then performed using the Luna Universal One-Step RT-qPCR Kit, adding 1 µg of RNA per reaction, with *gndf* gene expression levels normalized to the housekeeping gene GAPDH. Experiments were conducted in triplicate.

### 2.11 Cell viability, MAO-B and ROS tests

To evaluate the effects of overexpressing *gdnf* within an *in vitro* Parkinson’s disease model, we performed three cellular function tests. These tests aimed to determine whether *gdnf* overexpression could improve cell viability and function in cells compromised by MPTP treatment.

Cell viability was evaluated with LDH assay. This assay was critical in determining whether cell proliferation could be enhanced following *gdnf* overexpression in the neurotoxin-challenged cells.

The enzymatic activity of MAO-B was quantified using the Monoamine Oxidase (MAO) Assay Kit (ab241031), following the manufacturer’s instructions. We quantified the enzymatic activity of MAO-B to determine whether CRISPRa modulated its activity and to confirm an increase in its activity with increasing concentrations of MPTP. A density of 45,000 cells per well was established in a 24-well microplate with DMEM supplemented with 10% FBS. Post a 24-h incubation at 37°C and 5% CO_2_, cells were exposed to MPTP and subsequently transfected with either Lipofectamine 3,000^®^+ P3000 reagent or the nanobioconjugates to induce the overexpression of *gdnf*. Cellular homogenization followed in Assay Buffer XVIII/MAO Assay Buffer, and supernatants were collected after centrifugation at 10,000 xg. To precisely quantify MAO-B activity, either 10 µM clorgyline working solution or MAO-B substrate was added. Fluorescence was measured at 535/587 nm excitation/emission with a FluoroMax 4 spectrofluorometer (HORIBA Scientific, Piscataway, NJ, United States) at the 60-min mark.

Reactive oxygen species (ROS) visualization (i.e., hydroxyl and peroxyl radicals) was performed using the Cellular Reactive Oxygen Species Detection Assay Kit (Deep Red Fluorescence) (ab186029) to identify, following the manufacturer’s instructions. This test was conducted to qualitatively determine if there was a reduction in oxidative stress by inducing the overexpression of *gdnf* both in the model of cells treated with 500 μM and 1.25 mM MPTP. 70,000 cells were seeded per well in a 12-well microplate coated with poly-d-lysine at a 50 μg/mL concentration, using DMEM medium supplemented with FBS and P/S. Subsequently, cells were treated as previously described and imaged using confocal microscopy with an Olympus FV1000 microscope and a 40X objective. The specific excitation/emission wavelengths (nm) for ROS detection were set at 650/675 nm. Analysis was done on 10 images per sample, with a minimum of 6 cells analyzed per image, using Fiji^®^ software for image quantification.

### 2.12 Statistical analyses

The statistical significance of differences between experimental groups was evaluated using a One-Way ANOVAs, followed by Tukey’s multiple comparisons test as implemented in GraphPad Prism Software version 9.3 (GraphPad Software Inc, Boston, MA, United States). The threshold for statistical significance was set at *p*-value <0.05.

## 3 Results and discussion

### 3.1 Magnetic nanoparticle physicochemical characterizations

**FIGURE 1:**
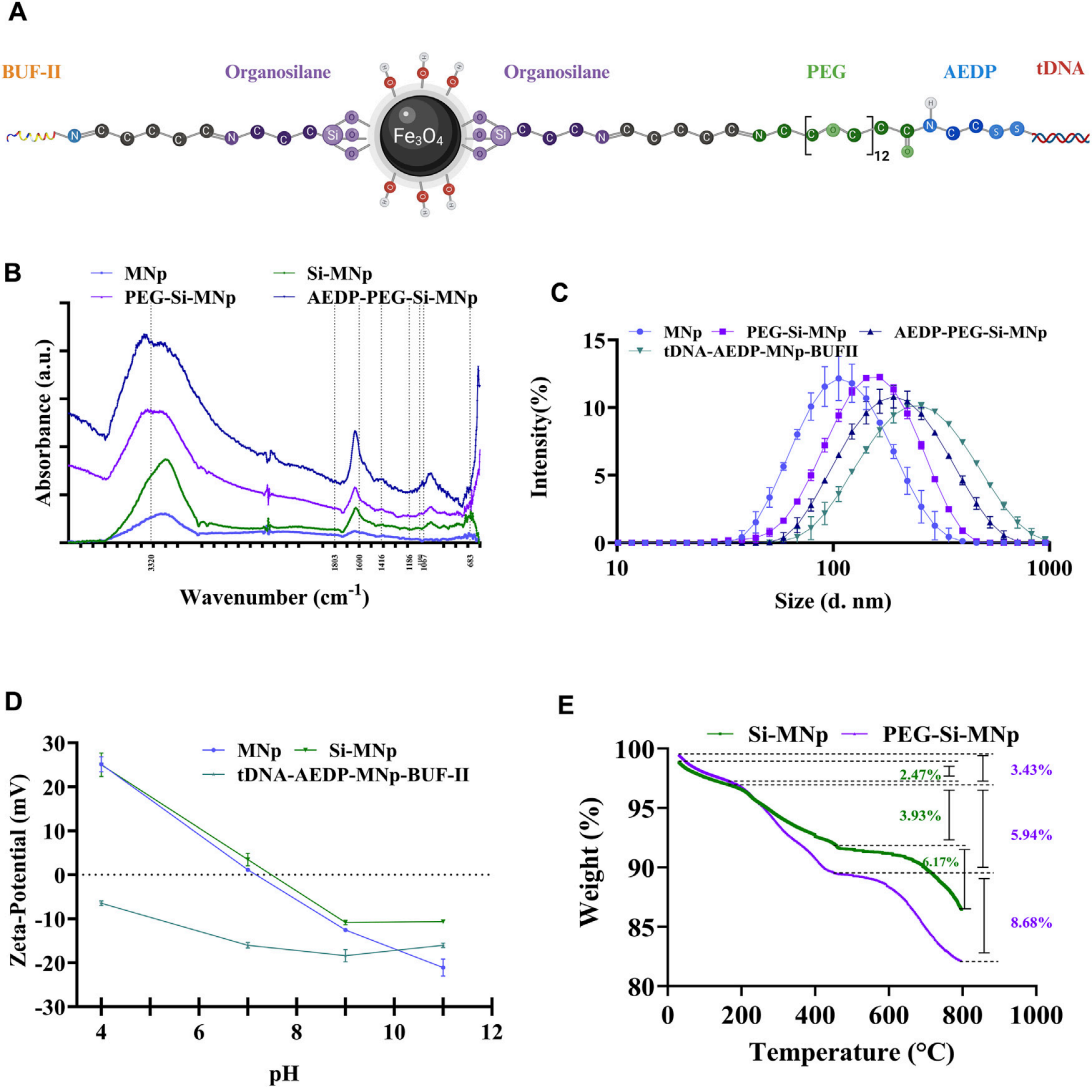
Physicochemical characterizations of nanobioconjugates. **(A)** Chemical structure of the nanovehicle. **(B)** FTIR spectra of MNP, Si-MNP, PEG-Si-MNP, and AEDP-PEG-Si-MNP nanoconjugates. **(C)** Hydrodynamic size distribution of MNPs, Si-MNP, PEG-Si-MNP, AEDP-PEG-Si-MNP and tDNA-AEDP-MNP-BUFII nanobioconjugate. **(D)** Zeta potential of the nanoconjugates. **(E)**. TGA thermograms of nanoconjugates.

We started by validating and evaluating the conjugation of PEG to nanoparticles through thermogravimetric analysis (Figure 1E). Our findings revealed an initial weight loss (20 °C–150 °C) of 2.47% and 3.43% for the Si-MNP and PEG-Si-MNP conjugates, indicative of sample dehydration. Additionally, we observed a more significant weight loss between 400°C and 800°C of 6.17% for Si-MNP and 8.68% for PEG-Si-MNP, which is consistent with previous findings (Ebadi et al., 2023) and attributed to the decomposition of immobilized surface molecules, including Si and PEG. At temperatures above 400 °C, magnetite can decompose into ferric oxide (Ebadi et al., 2023) and shows the transition from its crystalline structure to maghemite or hematite (Jafari et al., 2015; Yamanaka et al., 2022).

We performed hydrodynamic diameter measurements to corroborate successful functionalization (Figure 1C). We found average diameters expanding from 96 nm (polydispersity index (PI) of 19%) for MNPs to 129 nm (PI of 20%) post-silanization. Upon conjugation with AEDP (AEDP-MNP), the average diameter increased to 170 nm with a PI of 24%. For the final tDNA-AEDP-MNP-BUF-II nanobioconjugate, the average hydrodynamic diameter was 221 nm with a PI of 30%. The increase in particle size and PI with each functionalization step is indicative of a successful layering of the compounds on the nanoparticle surface.

The internalization of nanoparticles occurs through clathrin and claveola-mediated pathways, macropinocytosis, or cholesterol-dependent mechanisms (Francia et al., 2020). Notably, nanoparticle size is crucial for cellular internalization, and in this case, the size profile indicates suitability for genetic material delivery, supported by the inclusion of BUF-II, a peptide known for endosomal escape capabilities (Chen et al., 2016; Mazumdar et al., 2021). The actual size of the magnetic nanoparticles is likely smaller than the hydrodynamic diameter indicates, with measurements potentially reflecting nanoparticle aggregates rather than individual nanoparticles. It is posited that the MNPs may range between 10 and 15 nm, sizes conducive to nuclear localization (Mohammadi et al., 2021), with smaller nanoparticles having a greater likelihood of reaching the cell nucleus (Oh et al., 2011).

We then proceeded to carry out Fourier-transform infrared (FTIR) spectroscopy to confirm the surface chemistry of the nanoconjugates (Figure 1B), where characteristic peaks at 683 cm^−1^ for the Fe-O vibration of magnetite (Roacho-Pérez et al., 2020) and at 1,067 cm^−1^ for the tension vibration of the Si-O (Si-MNP) and at 1,100 cm^−1^ for the C-O bond upon conjugation with the organosilane were identified (Widjonarko et al., 2014). The vibrations at 3,320 cm^−1^ and 1,186 cm^−1^ verified the presence of -N-H and C-O-C groups from PEG (Hamdy et al., 2021), and a C=O bond peak at 1803 cm^−1^ in the AEDP-MNP spectrum marked the conjugation with AEDP (Abdulkadir et al., 2016).

We performed a Zeta potential analysis to know the surface charge of the nanoparticles at different pH levels. This showed significant variance, with potential ranging from 20 mV to 30 mV under low pH conditions and from −20 mV to −30 mV under high pH conditions (Soares et al., 2019; Markhulia et al., 2021), attributed to the presence of surface hydroxyl groups. These findings, detailed in Figure 1D, elucidate the zeta potential trends for both MNPs and Si-MNP conjugates across pH values of 4, 10, and 12. Additionally, it was discerned that magnetite nanoparticles attain their isoelectric point at physiological pH. The tDNA-AEDP-MNP-BUFII nanobioconjugates exhibited a zeta potential of −16 mV at physiological pH, which, while indicating potential cell membrane repulsion, does not preclude efficient internalization through endocytosis (Bai et al., 2018). Negatively charged nanoparticles have been shown to cross cellular membranes effectively (Ikeda et al., 2021), suggesting the suitability of these nanobioconjugates as delivery systems despite the negative charge from DNA conjugation.

### 3.2 Magnetic nanoparticle endosomal escape

**FIGURE 2:**
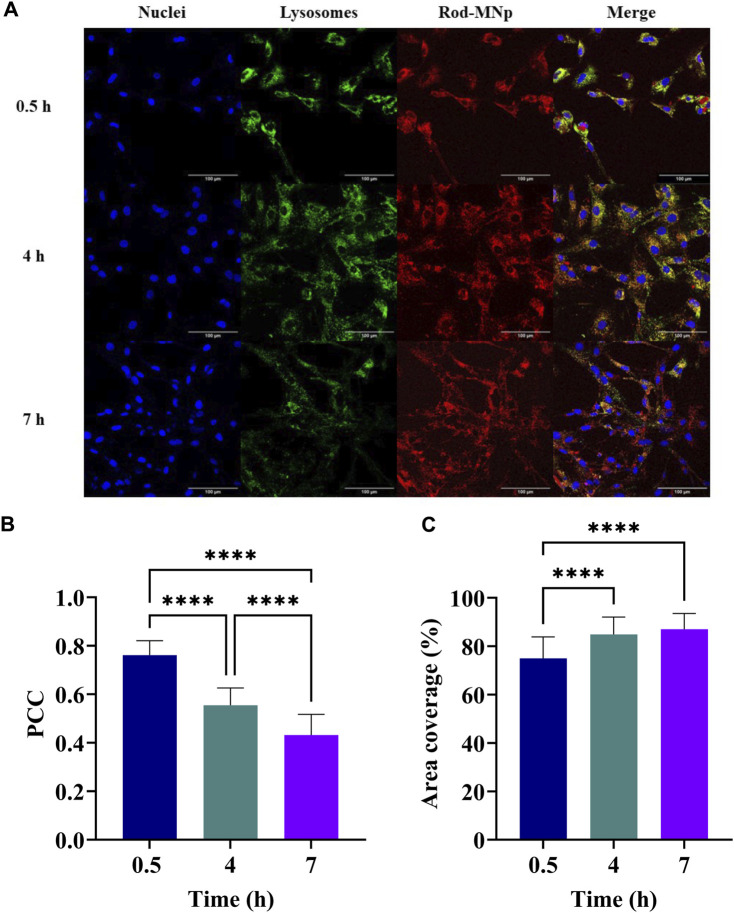
Endosomal escape of the Rhodamine-B-labeled nanobioconjugates. **(A)** Confocal microscopy images showing internalization of Rhodamine-B-labeled nanobioconjugates in a mixed culture of rat cells (astrocytes, microglia, and neurons). Nuclei were labeled with Hoechst dye (Blue). Endosomes were labeled with LysoTracker Green^®^. Scale bar 100 μm. **(B)** Pearson correlation coefficient (PCC) between nanoparticles and endosomes. 0.5 h: 0.761 ± 0.06, 4 h: 0.554 ± 0.071, 7 h: 0.432 ± 0.085. **(C)** Area covered by the Rhodamine-B-labeled nanobioconjugates. 0.5 h: 74.98% ± 8.92%, 4 h: 84.93% ± 7.113%, 7 h: 87.06% ± 6.447%. ANOVA and Tukey’s test were conducted to determine statistical significance. ** indicates *p*-values <0.01, *** *p*-values <0.001, and **** *p*-values <0.0001.

We evaluated whether our gene editing delivery vehicle escapes the endosome and thus successfully translocates into the cell. We evaluated colocalization between the Rhodamine-B-labeled nanobioconjugates and the endosomes labeled with LysoTracker. Our analysis revealed significant findings regarding the endosomal escape of the nanobioconjugates. Initial Pearson Correlation Coefficient (PCC) values at 0.5 h indicated substantial endosomal entrapment with a PCC of 0.761 ± 0.06. Over time, the PCC decreased to 0.554 ± 0.071 at 4 h and further to approximately 0.432 ± 0.085 after 7 h, as detailed in Figure 2B. This progressive decrease is consistent with enhanced endosomal escape over time and aligns with previous research on similar nanoconjugates containing BUF-II, a peptide known for its efficacy in membrane translocation due to arginine residues and proline hinge (Perez et al., 2019). The increase in cellular internalization is also facilitated by the protonation of tertiary and primary amines on the nanoconjugate, aiding its uptake into cells (Freeman et al., 2013).

These results are consistent with previous studies using magnetite nanobioconjugates for siRNA delivery, which were tested in research to treat Alzheimer’s disease (Lopez-Barbosa et al., 2020). Furthermore, endosomal escape has been shown to vary across different cell lines, with one study reporting a PCC of 0.4 at 4 h when using magnetite nanoparticles with BUF-II for plasmid release (Ramírez-Acosta et al., 2020). The coverage area of the nanobioconjugates within the cells, as shown in Figure 2C, also increased significantly throughout the exposure period, reaching 87.06% ± 6.447% % at 7 h. This suggests a successful cellular uptake of the nanoparticles.

Endosomal escape is a critical parameter for delivery systems that aim to transfect and release genetic material inside cells. Given the increased endosomal escape and cellular membrane translocation over time, we can affirm that the synthesized nanobioconjugate exhibits promising potential as a delivery system for genetic material.

### 3.3 *In vitro* Parkinson’s disease model

**FIGURE 3:**
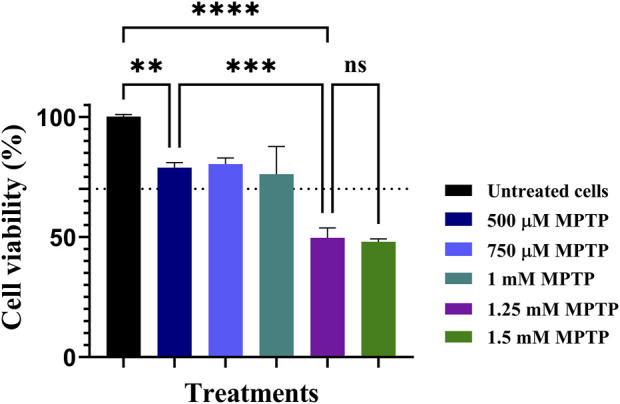
Evaluation of cell viability in MPTP Parkinson’s disease model at different MPTP concentrations. ANOVA and Tukey’s test were conducted for statistical analysis. ** indicates *p*-values <0.01, *** *p*-values <0.001, and **** *p*-values <0.0001.The dashed line indicates a cell viability of 70%.

To evaluate the efficiency and pertinence of the system to correct the disruption in a biological pathway associated with PD by overexpressing *gdnf*, it is necessary to establish an appropriate cellular PD model and evaluate its pertinence. Here, we used MPTP (1-methyl-4-phenyl-1,2,3,6-tetrahydropyridine hydrochloride), which had been previously used as a toxin to induce PD symptoms in cellular systems (Goloborshcheva et al., 2022). We quantified the cytotoxic effects of MPTP on a mixed co-culture of neurons, astrocytes, and microglia 24 h after exposure (Figure 3). The cell viability remained relatively stable at 78.893% ± 2.074%, 80.383% ± 2.503%, and 76.155% ± 11.52% for MPTP concentrations of 500 μM, 750 μM, and 1 mM, respectively, suggesting a lack of significant cytotoxicity at these levels. In contrast, a marked reduction in viability was observed at concentrations of 1.25 mM and 1.5 mM MPTP, where viability dropped to 49.612% ± 4.145% and 47.959% ± 1.248%, respectively, indicating cytotoxic effects.

These findings are consistent with previous studies, which has established that MPTP concentrations above 1 mM lead to a notable decrease in cell viability ranging between 25% and 30%, whereas concentrations below this threshold are not associated with cytotoxicity (Gao et al., 2013). Our results corroborate this threshold effect, highlighting the non-cytotoxic nature of lower MPTP concentrations and the toxic potential at 1.25 mM and higher concentrations. In accordance with what has been previously reported in the literature, we found that at concentrations greater than 1 mM, cellular death exceeds 48%, and cellular viability decreases as MPTP concentration increases, showing that the cells are sensitive to MPTP.

The MPTP-induced model is a well-recognized method for replicating Parkinson’s disease characteristics *in vitro* and *in vivo*, as it simulates the neuroinflammation, cell death, oxidative stress, and mitochondrial dysfunction observed in PD pathology (Supplementary Figure S12) (Miyazaki and Asanuma, 2020; Goloborshcheva et al., 2022; Wang et al., 2024a). Through the overexpression of *gdnf*, it is possible to mitigate these toxic effects. GDNF has been reported to safeguard mitochondrial functional activity and structure (Mitroshina et al., 2019), to bolster the survival of a wide array of cell types beyond neurons (Sun et al., 2020) and it presents a significant therapeutic potential for treating neuroinflammation (Singh et al., 2023), reinforcing its value in PD treatment strategies. Considering our results, we used two MPTP-induced PD models at concentrations of 500 μM and 1.25 mM for the rest of this study. From now on, we will refer to these MPTP-induced models as 500 µM (IC20) and 1.25 mM (IC50) MPTP-treated cells.

### 3.4 Overexpression validation

We relied on RT-qPCR to confirm that we successfully overexpressed *gdnf*. We measured *gdnf* expression in both MPTP-induced model cells, at 500 μM and 1.25 mM MPTP, and at two time points, 24 and 48 h after treatment with the *gdnf*-CRISPRa nanobioconjugate.

**FIGURE 4:**
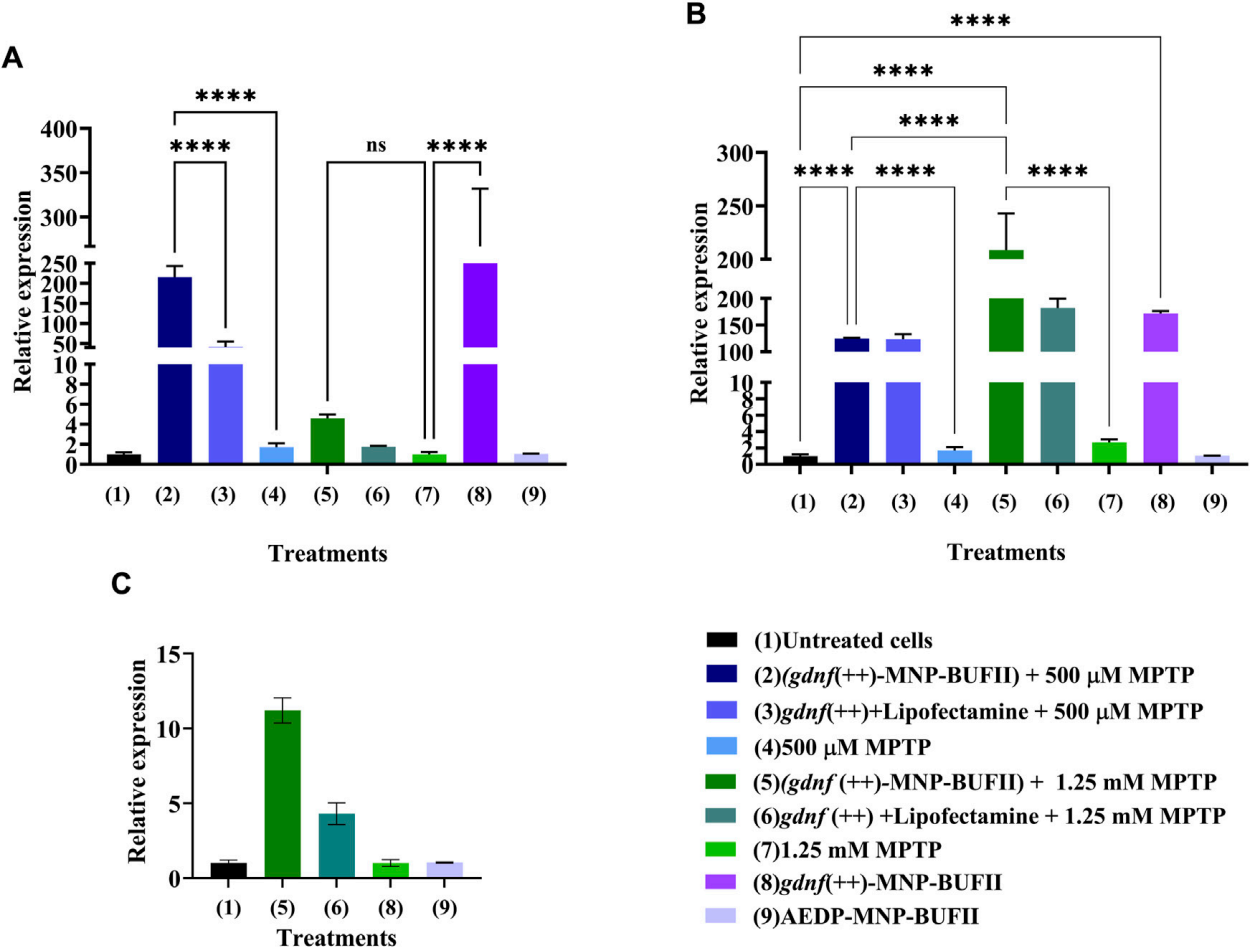
Validation of *gdnf* overexpression using RT-qPCR. **(A)** Relative *gdnf* expression after 24 h of the delivery of *gdnf*-CRISPRa nanobioconjugate. **(B)** Relative expression after 48 h of the delivery of the *gdnf*-CRISPRa nanobioconjugate. **(C)** Relative expression after 24 h of the delivery of *gdnf*-CRISPRa nanobioconjugate (GAPDH Ct values were calculated by the geometric mean of Ct values at 24 and 48 h). The GAPDH gene was used as a housekeeping gene. ANOVA and Tukey’s test were conducted for statistical analysis. ** indicates *p*-values <0.01, *** *p*-values <0.001, and **** *p*-values <0.0001.

The MNP vehicle worked effectively, delivering CRISPRa elements to treated cells and increasing *gdnf* expression (Figure 4). This was evident in the 500 μM MPTP-treated cells after 24 h and 48 h of treatment, where we saw an increase in *gdnf* expression of 214 -fold (214.97 ± 28.36) and 208 -fold (208.82 ± 34.10) times higher than our control, untreated cells. The comparable levels of gene activation at both 24 and 48 h imply that the maximum level of gene upregulation was reached early and maintained. This notable increase in expression levels indicates that the delivery vehicle carrying the CRISPRa elements made it properly inside the cell and effectively caused the overexpression of *gndf*. This result confirms previous findings from (Arango et al., 2023), where a similar nanostructured system was used and significantly increased gene expression.

Our results also indicate that the MNP nanobioconjugate we used is effective in delivering CRISPRa elements into the cells than a traditional Lipofectamine transfection. We found that cells treated with lipofectamine for transfection showed lower levels of *gdnf* expression at 24 and 48 h (Figure 4). The most dramatic difference can be observed after 24 h when lipofectamine-treated cells only showed a 41-fold (41.538 ± 13.412) increase in expression (Figure 4A).

We observed low expression levels of *gdnf* in 1.25 mM MPTP-treated cells in both the nanobioconjugate and lipofectamine treatments at 24 h. The expression of our housekeeping gene, GAPDH, could drive these results. It is documented that GAPDH, traditionally used as a housekeeping gene, plays a role in cell death pathways, and is upregulated under oxidative stress conditions, causing its expression to increase in cellular contexts marked by apoptosis (Nakajima et al., 2017). The association of GAPDH with cell death could be due to cellular toxicity triggering its nuclear translocation, where it may activate apoptotic mechanisms (Zhai et al., 2014). To address this issue, the relative expression values for cells treated with 1.25 mM MPTP were recalculated using the geometric mean of Ct values of GAPDH at 24 and 48 h (Figure 4C). This is meant to make the final results less sensitive to temporary increases in GAPDH reference levels. Upon recalculating the Ct values of GAPDH, the data indicated an 11-fold (11.204 ± 0.835) increase in *gdnf* expression after 24 h of treatment with the *gdnf*-CRISPRa nanobioconjugate. While it remains an approximation, this method allows us to correct for the fact that the reference gene is most likely altered in cells subjected to the aggressive conditions we had to use to simulate PD symptoms at 1.25 mM MPTP. At such high concentrations of MPTP, it is difficult to find routes that do not become dramatically altered after 24 h or 48 h.

### 3.5 Cell viability, MAO-B and ROS tests

To ascertain the effect of *gdnf* overexpression, we studied relevant aspects of cellular function in multiple experimental conditions to assess whether this CRISPRa treatment could rescue the cells from the cellular effects induced by MPTP. In summary, we had the following experimental conditions: as controls, we had healthy untreated cells and healthy cells treated with the *gdnf*-CRISPRa nanobioconjugate. Additionally, for 500 μM and 1.25 mM MPTP-treated cells, function was compared in cells before and after treatment with the *gdnf*-CRISPRa nanobioconjugate. Finally, we tested the effect of using our *gdnf*-CRISPRa nanobioconjugate *versus* traditional transfection with lipofectamine, adding this final treatment to each 500 μM and 1.25 mM MPTP-treated cells.

**FIGURE 5:**
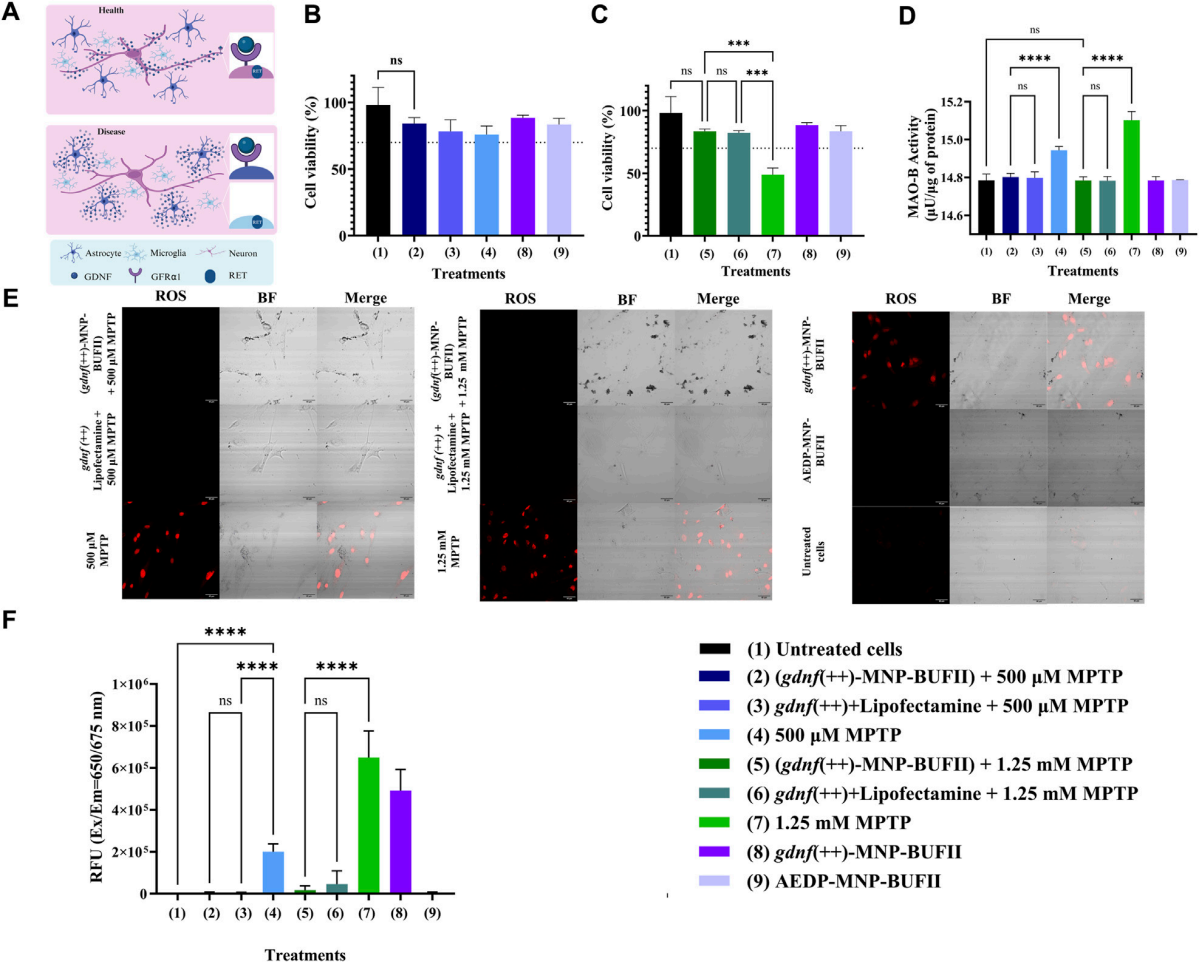
Cell viability, MAO-B and ROS tests. **(A)** GDNF and its relationship with GFRα1 and RET (scheme based on (Duarte Azevedo et al., 2020): GDNF forms a complex with the GFRα1 receptor, which in turn binds to RET, thereby activating signaling pathways associated with cell survival, differentiation, and growth. Under conditions of disease, when neurons die, the GDNF-GFRα1 receptor complex forms in astrocytes, and the RET receptor is found in microglia. **(B)** Cell viability with different treatments (MPTP 500 µM model). **(C)** Cell viability with different treatments (1.25 mM MPTP model). **(D)** MAO-B activity under different treatments. **(E)** Representative confocal images showing the ROS levels with different treatments. Scale bar 40 μm. **(F)**Quantification of the fluorescence associated with ROS levels (RFU: Relative Fluorescence Units). ANOVA and Tukey’s test were conducted for statistical analysis. ** indicates *p*-values <0.01, *** *p*-values <0.001, and **** *p*-values <0.0001. The dashed line indicates a cell viability of 70%.

*Cell viability*: cell viability in a mixed co-culture of neurons, astrocytes, and microglia cells was evaluated for 48 h (Figure 5B). As expected, at the higher concentration of MPTP (1.25 mM), cell viability dropped to 48.85% ± 5.390%, in line with earlier findings (Gao et al., 2013). We observed, however, a marked improvement in cell viability following *gdnf*-CRISPRa nanobioconjugate treatment, where cellular viability significantly increased to 83.486% ± 1.870 (compared to 34%). The effects of treating cells with MPTP at concentrations of only 500 μM, were not significant across the different treatments (Figure 5C). Importantly, we found that increasing *gdnf* expression with CRISPRa had an effect on recovering cellular viability after exposure to elevated MPTP concentrations.

Previous research has documented GDNF’s protective role against oxidative stress generated by neurotoxins such as MPTP, although the precise mechanisms remain to be fully elucidated. GDNF is thought to regulate endogenous antioxidant molecules and suppress the production of pro-oxidative compounds (Littrell et al., 2013) and is involved in critical signaling pathways related to cell survival and proliferation, including RET, PI3K/Akt, and MAPK/ERK (Li et al., 2022). This effect was evident in our results since proliferation and recovery were evident in cells treated with *gdnf*-CRISPRa nanobioconjugate.

*MAO-B activity*: We quantified MAO-B activity because this enzyme complex is responsible for metabolizing MPTP to MPP+. Therefore MAO-B activity is expected to decrease if cells affected by MPTP are recovering after CRISPRa treatment to overexpress *gdnf*. As expected, we found that *gdnf* overexpression led to a decrease in MAO-B activity (Figure 5D), intricately linked to the metabolism of MPTP to MPP+. The activity of MAO-B in cells exposed to 500 µM MPTP was 14.943 ± 0.019 μU/μg of protein. In cells exposed to 1.25 mM MPTP, the activity was 15.102 ± 0.0457 μU/μg of protein. For cells exposed to 1.25 mM MPTP and treated with *gdnf*-CRISPRa nanobioconjugate, the activity was 14.783 ± 0.0196 μU/μg of protein. In cells exposed to 500 µM MPTP and treated with *gdnf-*CRISPRa nanobioconjugate, the activity was 14.801 ± 0.0199 μU/μg of protein, and in untreated cells, the activity was 14.784 ± 0.033 μU/μg of protein.

Our results suggest that while GDNF does not directly inhibit MAO-B, its overall protective effects on cells, particularly concerning oxidative stress and mitochondrial function, could be associated with a mitigating impact on MAO-B activity. This relationship may be influenced by the interaction between GDNF and the NF-κB transcription factor, as well as the PI3K pathway (Machado et al., 2016).

*Reactive oxygen species (ROS) production*: When cells are exposed to MPTP, it results in the inhibition of complex I in the mitochondria, which leads to the production of reactive oxygen species and, thus, cellular oxidative stress and damage (Choi et al., 2008). If CRISPRa treatment to overexpress *gdnf* is working to correct the cellular damage caused by MPTP, we would thus expect to see a decrease in ROS production as this gene generates protection against cellular oxidative stress. We found a reduction in fluorescence associated with ROS for 500 μM and 1.25 mM MPTP-treated cells after we triggered *gdnf* overexpression (Figures 5E,F). We found the same effect for both lipofectamine and *gdnf*-CRISPRa nanobioconjugate delivery.

The reduction in ROS we observed is consistent with GDNF’s recognized neuroprotective role, highlighting its use against neurotoxic stress (Duarte Azevedo et al., 2020). GDNF, upregulated in glial cells during stress, plays a pivotal role in neuroprotection and repair, acting through its receptor complex involving GFRα1 and RET (5A), which triggers critical intracellular pathways for neuronal health (Duarte Azevedo et al., 2020). Its ability to influence neuroinflammation also explains GDNF’s role in counteracting oxidative stress induced by neurotoxic compounds like MPTP (Duarte Azevedo et al., 2020).

However, a contrasting outcome is also presented in Figures 5E,F—an increase in ROS levels in healthy cells treated with the *gdnf* -CRISPRa nanobioconjugate. This suggests that in the absence of MPTP, cellular receptors for GDNF, specifically GFRα1 and RET, may be underexpressed, preventing the formation of active GDNF-receptor complexes and leading to an accumulation of free GDNF (Figure 5A). This scenario is similar to high GDNF exposure conditions previously associated with adverse effects, including altered neurotransmitter homeostasis (Tenenbaum and Humbert-Claude, 2017). Consequently, the hypothesis emerges that the observed ROS increase is due to the excess free GDNF when receptor binding is limited, highlighting the intricate balance required in GDNF signaling for its neuroprotective properties to prevail.

GDNF is intricately linked to downstream signaling pathways such as the Akt/mTOR, MAPK/ERK, and NF-κB pathways (WANG et al., 2007; Kurtzeborn et al., 2019; Singh et al., 2023; Fei et al., 2024). These pathways are pivotal for neuronal survival, cell proliferation, and the attenuation of neuroinflammation. Therefore, activating these pathways associated with GDNF could help alleviate the cellular effects induced by neurotoxins utilized in Parkinson’s disease models.

It is important to note that the expression of *gdnf* has previously been associated with the development and progression of gliomas (Fielder et al., 2018; Yu et al., 2022). Therefore, chronic overexpression of GDNF could potentially lead to unintended adverse effects. Specifically, GDNF may contribute to the malignancy of gliomas by enhancing their invasive capabilities and promoting tumor cell proliferation. This underscores the importance of carefully regulating GDNF. However, our study shows that expression begins to decrease over time in cells treated with the *gdnf*-CRISPRa nanobioconjugate (Figures 4A,B).

Going forward, it will also be important to assess potential off-targets when using CRISPRa to overexpress *gdnf* in more complex and long-term models as off-target effects could impact this system’s safety. This will have to be evaluated *in vivo* to continue studying the potential of *gdnf* targeted gene expression modification as a treatment alternative for Parkinson’s disease.

## 4 Conclusion

In this study, we present evidence that CRISPRa-mediated overexpression of *gdnf* can mitigate cellular effects disrupted by neurotoxins such as MPTP, widely used as a Parkinson’s disease models. Our CRISPRa treatment’s success hinges on a nanostructured magnetite vehicle meticulously engineered and optimized for efficient delivery and controlled release of genetic material to targeted cells. Quantitative physicochemical characterizations confirm this nanoconjugate possesses all expected properties of an effective nucleotide delivery system. Moreover, quantification of *gdnf* relative expression demonstrates that the nanobioconjugate outperforms conventional methods for delivering CRISPRa DNA elements into cells. The achieved high transfection efficiency and evidence supporting *gdnf* overexpression’s potential to reverse cellular impairments caused by MPTP underscore the promise of CRISPRa.

Looking forward, quantifying *gdnf* overexpression in pathological contexts will be crucial, as well as validating efficacy and safety in more complex biological models, to compare outcomes with dopamine precursor use or deep brain stimulation. Furthermore, elucidating the relationship between *gdnf* expression levels and specific cellular metabolic activities will be critical for defining measurable outcomes.

## Data Availability

The original contributions presented in the study are included in the article/Supplementary Material, further inquiries can be directed to the corresponding author.
