# Supplementary material for: Assessment of CRISPRa-mediated gdnf overexpression in an In vitro Parkinson’s disease model
Source: Front Bioeng Biotechnol. 2024 Aug 8;12:1420183. doi: 10.3389/fbioe.2024.1420183 (PMC11338903; doi:10.3389/fbioe.2024.1420183)
Supplement: Supplementary file 1 [file DataSheet1.docx]

Supplementary Material

**Assessment of CRISPRa-mediated *gdnf* overexpression in an *In vitro* Parkinson's Disease model**

**Paula Guzmán-Sastoque^1^, Sebastian Sotelo^1^, Natalia Esmeral^1^, Sonia Luz Albarracín^2^, Jhon-Jairo Sutachan^2^, Luis H. Reyes^3^, Carolina Muñoz-Camargo^1^, Juan C. Cruz^1,3,*^, Natasha I. Bloch^1, *^**

^1^Biomedical Engineering Department, Universidad de los Andes, Bogotá, Colombia

^2^Nutrition and Biochemical Department, Pontificia Universidad Javeriana, Bogotá, Colombia

^3^Grupo de Diseño de Productos y Procesos (GDPP), Chemical and Food Engineering Department, Universidad de los Andes, Bogotá, Colombia

*** Correspondence:**

Juan C. Cruz

j[c.cruz@uniandes.edu.co](mailto:Jc.cruz@uniandes.edu.co)

Natasha I. Bloch
[n.blochm@uniandes.edu.co](mailto:n.blochm@uniandes.edu.co)


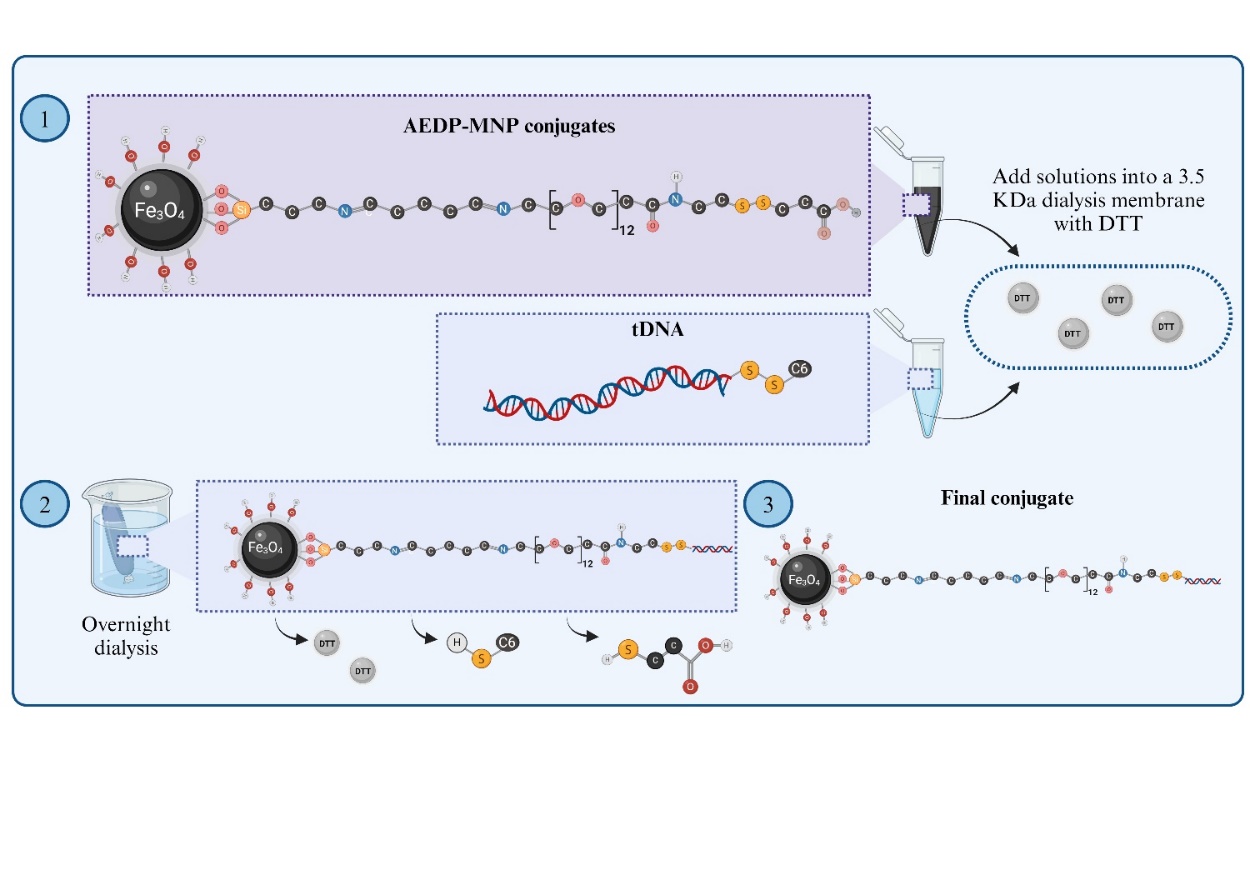


**Figure S1.** Representative scheme of tDNA conjugation to AEDP-PEG-MNP nanoconjugates by disulfide exchange reaction.


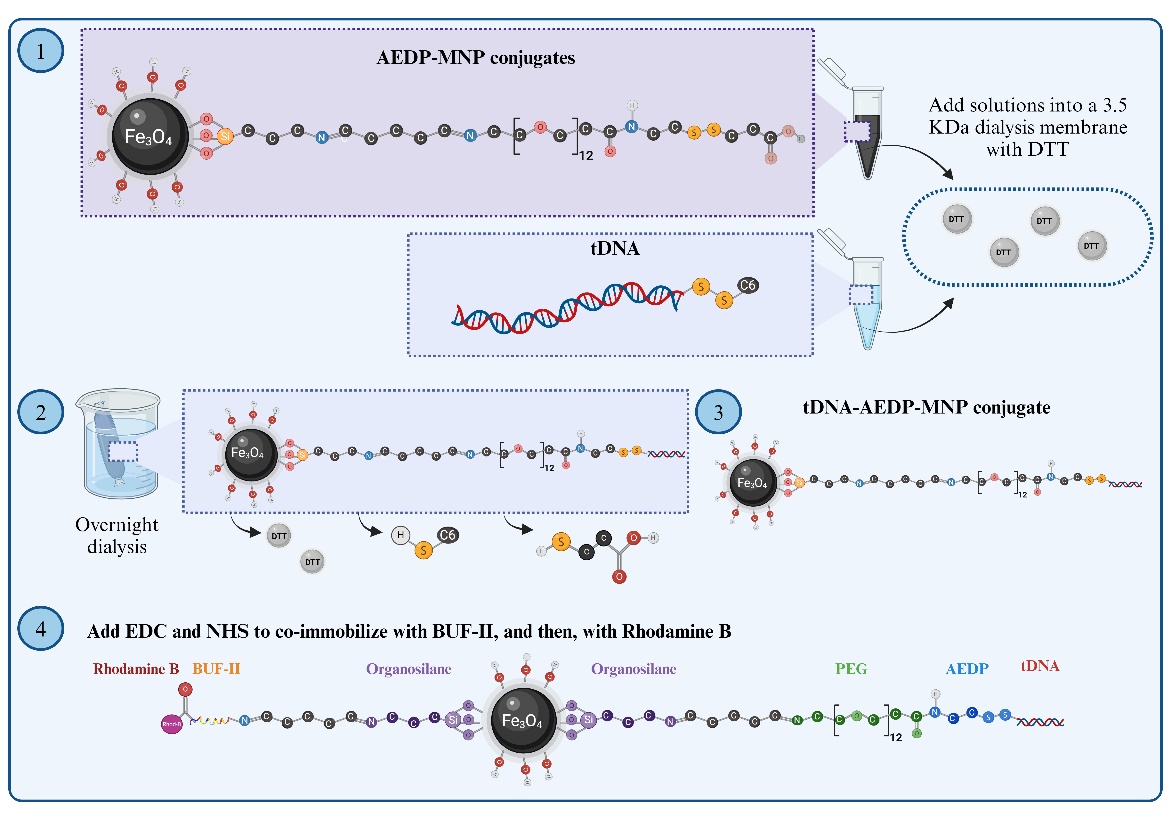


**Figure S2.** Representative scheme of tDNA fluorescent labeling and conjugation to AEDP-PEG-MNP nanoconjugates by disulfide exchange reaction.


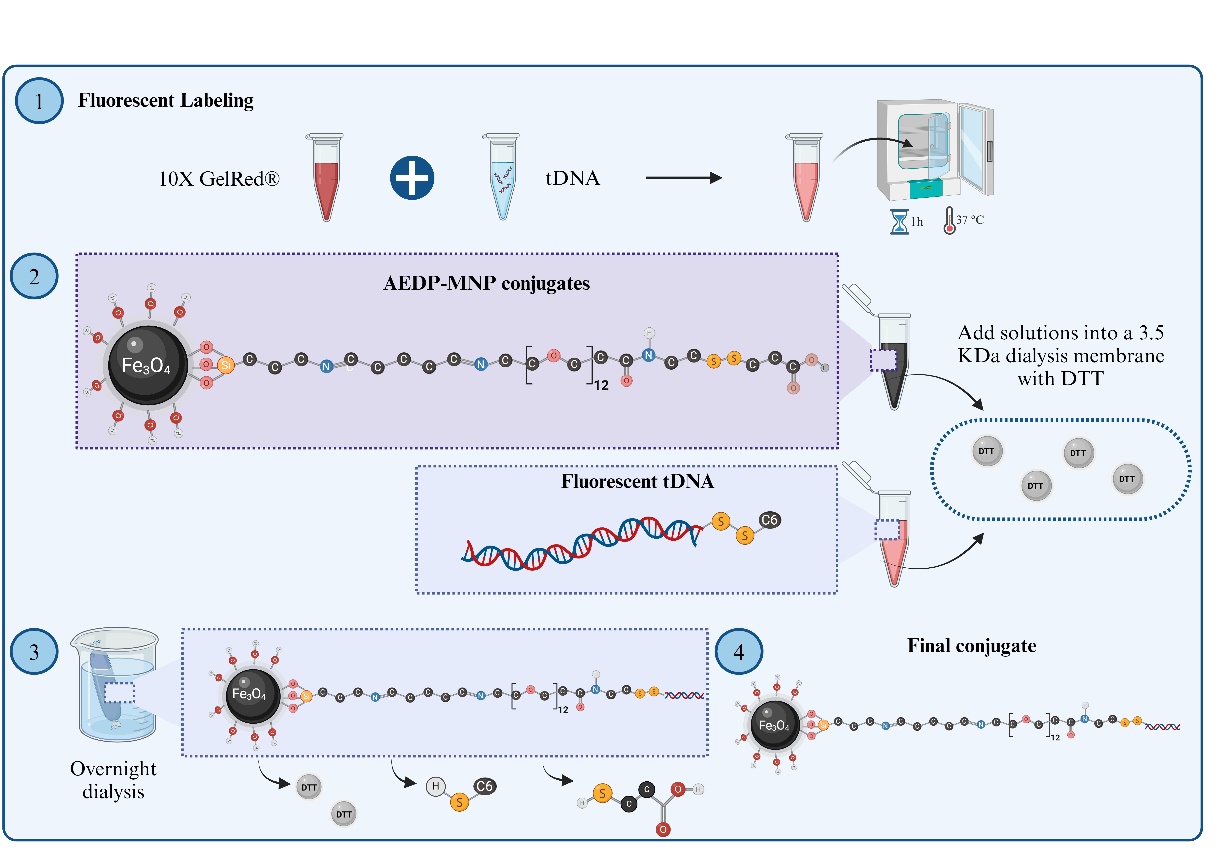


**Figure S3.** Representative scheme of tDNA conjugation to AEDP-PEG-MNP nanoconjugates by disulfide exchange reaction and fluorescent labeling with Rhodamine B.

**Table S1.** sgRNA sequences and parameters used to select the guides.

| **Guide** | **Sense** | **Species** | **Gene** | **FW** | **RW** | **Position TSS (transcription start site)** | **On target score** | **TI BI** | **TII BI** | **TIII BI** | **TI BII** | **TII BII** | **TIII BII** | **TIII BIII** | **TIII BIV** |
| --- | --- | --- | --- | --- | --- | --- | --- | --- | --- | --- | --- | --- | --- | --- | --- |
| *gdnf*-1 | Pos | Rat | *gdnf* | GATCGAAGATAATTTGTATATCAAGG | AAAACCTTGATATACAAATTATCTTC | -184 | 0.67 | 0 | 0 | 0 | 2 | 0 | 191 | 341 | 423 |
| *gdnf*-2 | Pos | Rat | *gdnf* | GATCGTATATCAAGGGGCAGCATCTG | AAAACAGATGCTGCCCCTTGATATAC | -185 | 0.52 | 0 | 0 | 0 | 0 | 0 | 176 | 286 | 536 |

CFD: Cutting Frequency Determination (CFD) is a score ranging from 0 to 1 that allows for the measurement of identity between the guide and any other genomic region, where 1 signifies complete identity.

BI: CFD = 1.0

BII: 0.2 ≤ CFD <1.0

BIII: 0.05 ≤ CFD <0.2

BIV: CFD <0.05

TI: Region relative to TSS of a protein-coding gene

TII: Region relative to TSS of a non-coding gene

TIII: All regions that are not in Level I-II

**Table S2.** Primers sequences.

| **Primer** | **Sequence** |
| --- | --- |
| Thiol-modified reverse | CGGAGGACCGAAGGAGCTAAC |
| *gapdh* forward | AGGGCTGCCTTCTCTTGTGACAAA |
| *gapdh* reverse | ATTCTCAGCCTTGACTGTGCCGTT |
| *gdnf* forward | TGGCTGTTCCGCGCTTCTTCTT |
| *gdnf* reverse | ACAAGCAGCCTGCCGAAGTT |


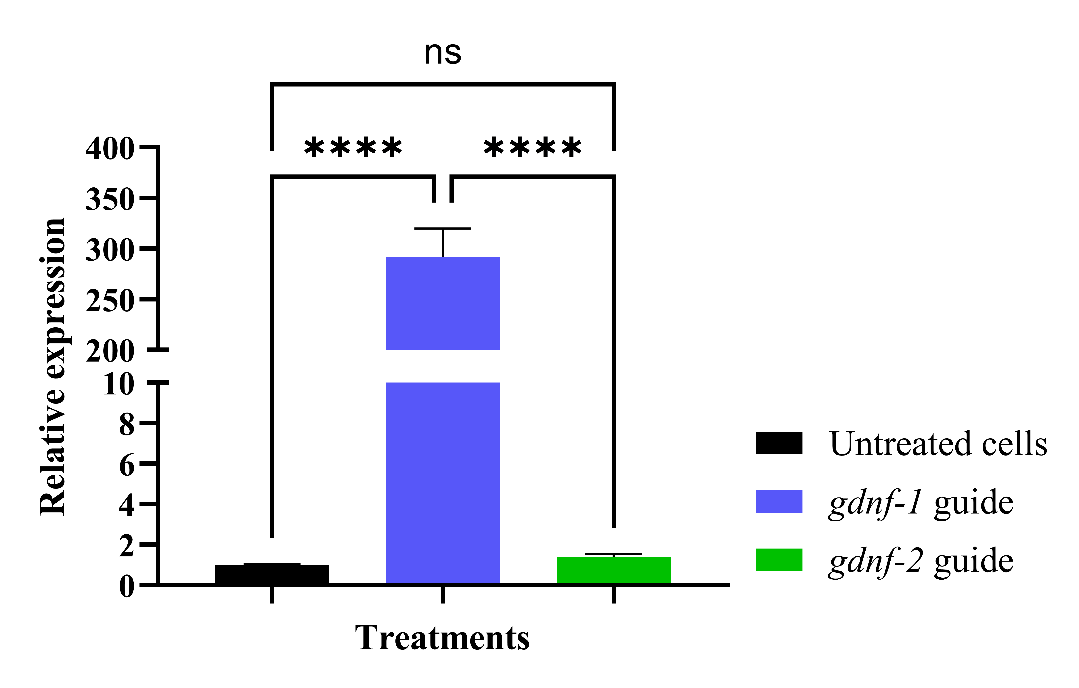


**Figure S4.** Selection guide: Relative expression after 48 h of the delivery of *gdnf.* The GAPDH gene was used as a housekeeping gene. ANOVA and Tukey’s test were conducted for statistical analysis, using 5% as a significance level. ** represents a significant difference with a p-value < 0.01, *** represents a significant difference with a p-value < 0.001, **** represents a significant difference with a p-value < 0.0001.


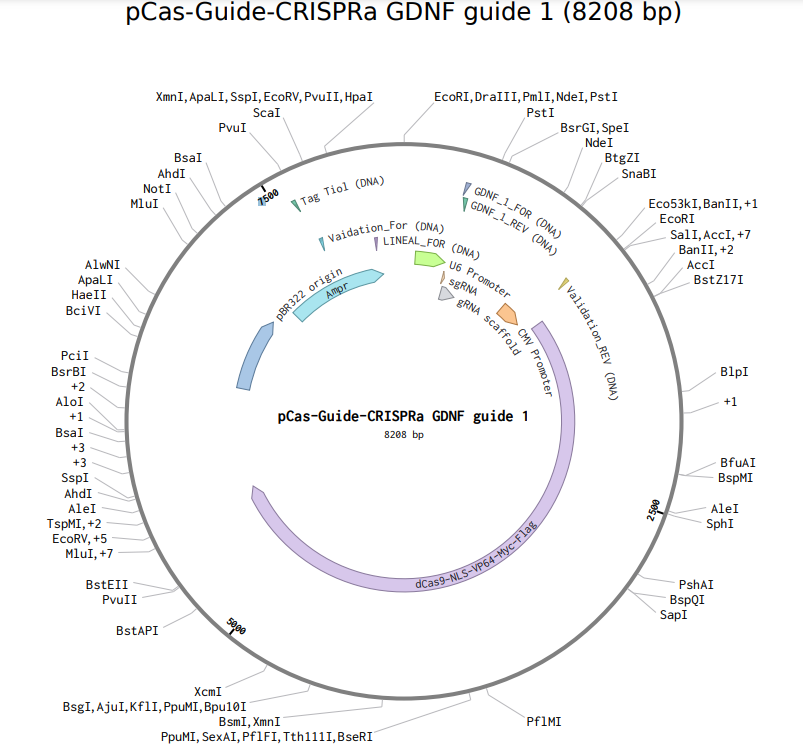


**Figure S5.** CRISPRa-*gdnf* 1 plasmid. Designed with Benchling [Biology Software].


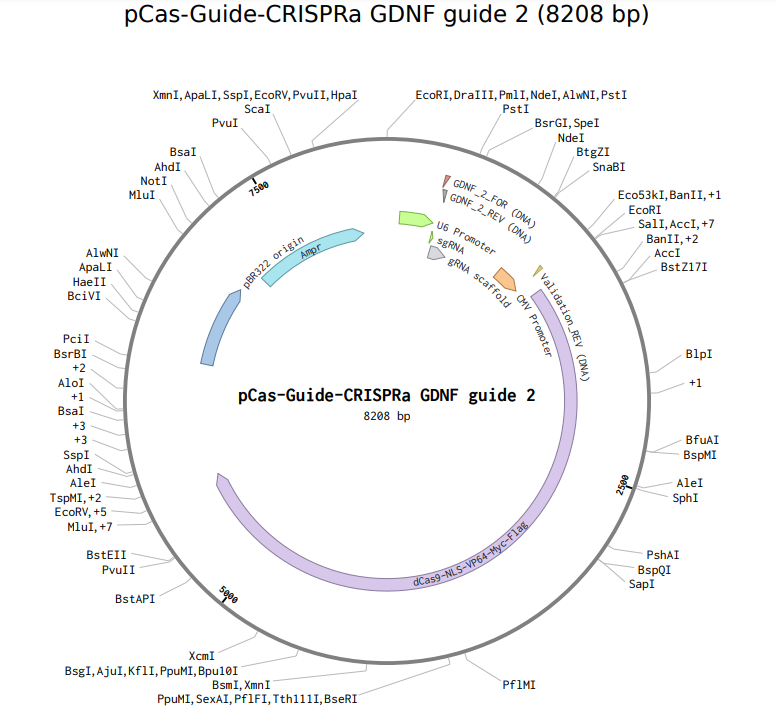


**Figure S6.** CRISPRa-*gdnf* 2 plasmid. Designed with Benchling [Biology Software].


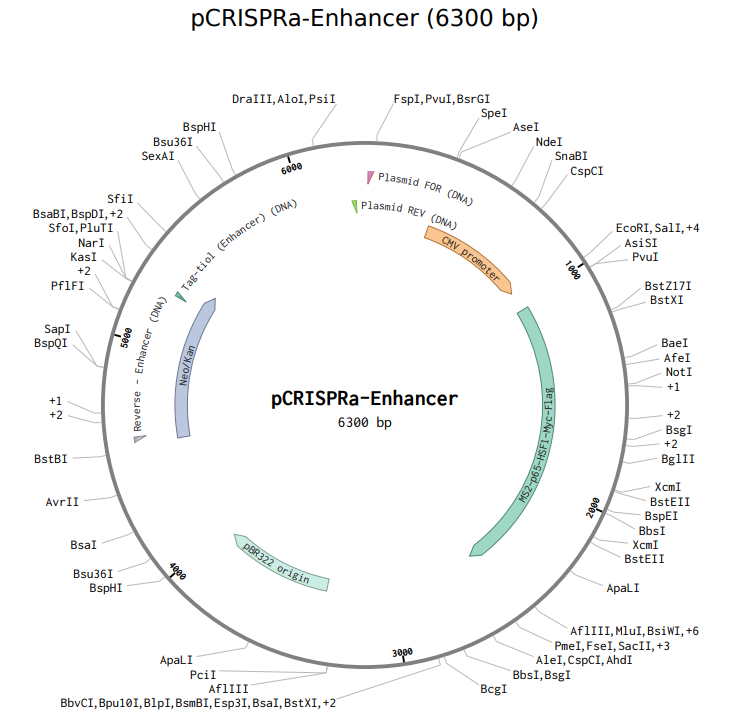


**Figure S7.** CRISPRa-Enhancer plasmid. Designed with Benchling [Biology Software].


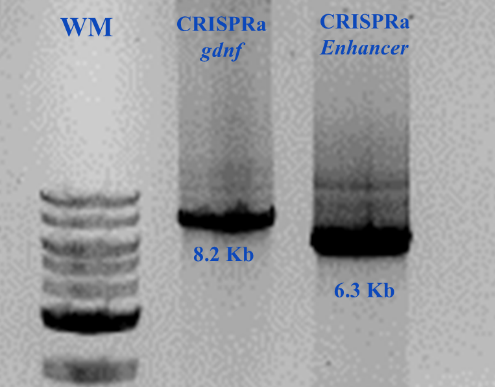


**Figure S8.** Electrophoresis gel confirmed the correct amplification and linearization of plasmids. Lane 1: Weight marker (WM). In lane 2 and 3, the correct linearization of CRISPRa-*gdnf* and CRISPRa-Enhancer is evident.


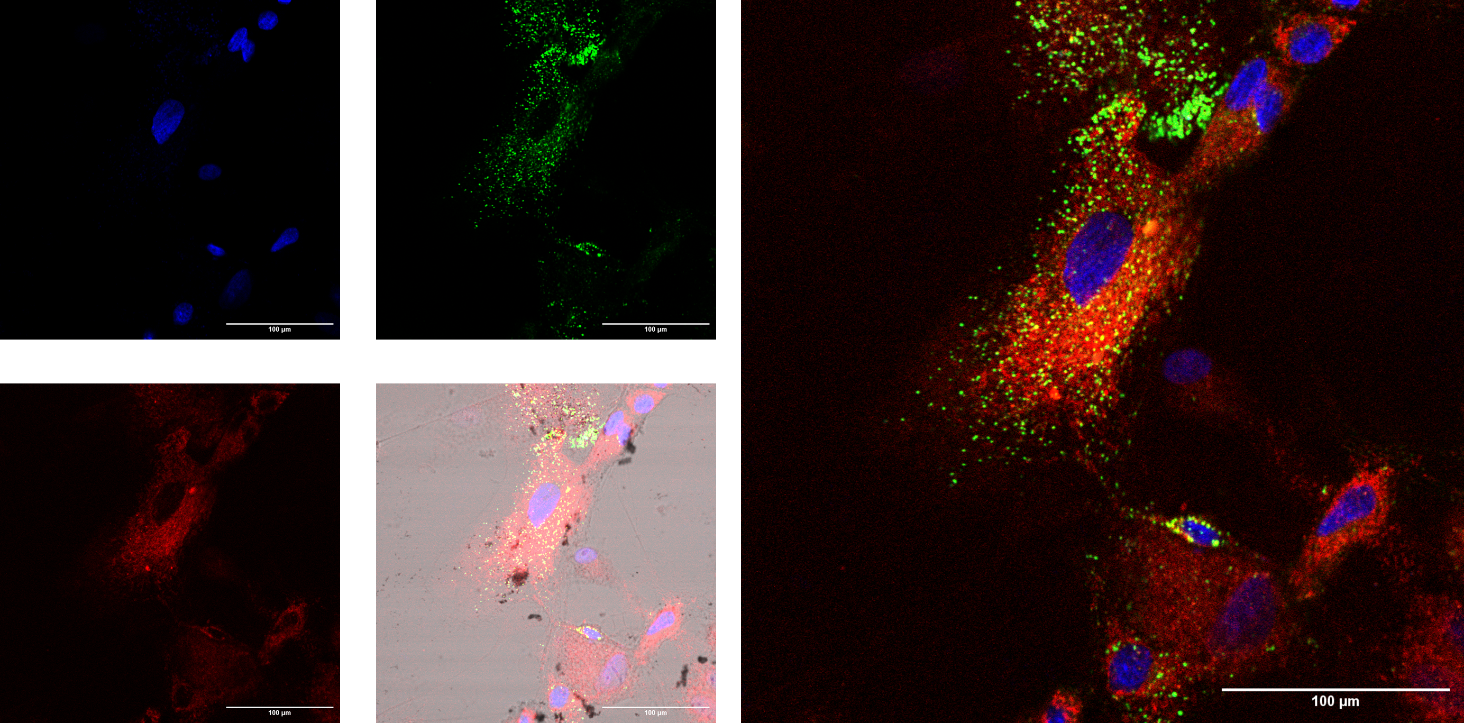


**Merge**

**BF**

**GelRed: *gdnf(*++)-MNP-BUFII -**

**Lysosomes**

**Nuclei**

**Figure S9.** Validation of the conjugation of the CRISPRa system to the nanoparticle with GelRed.


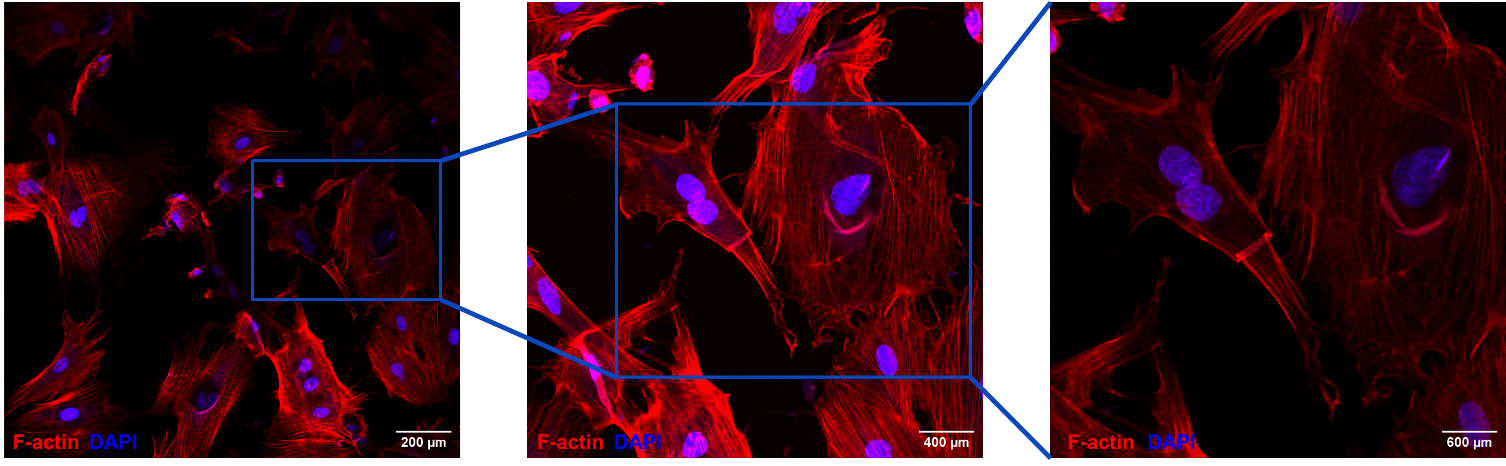


**Figure S10.** *In vitro* cell culture after 6 days.


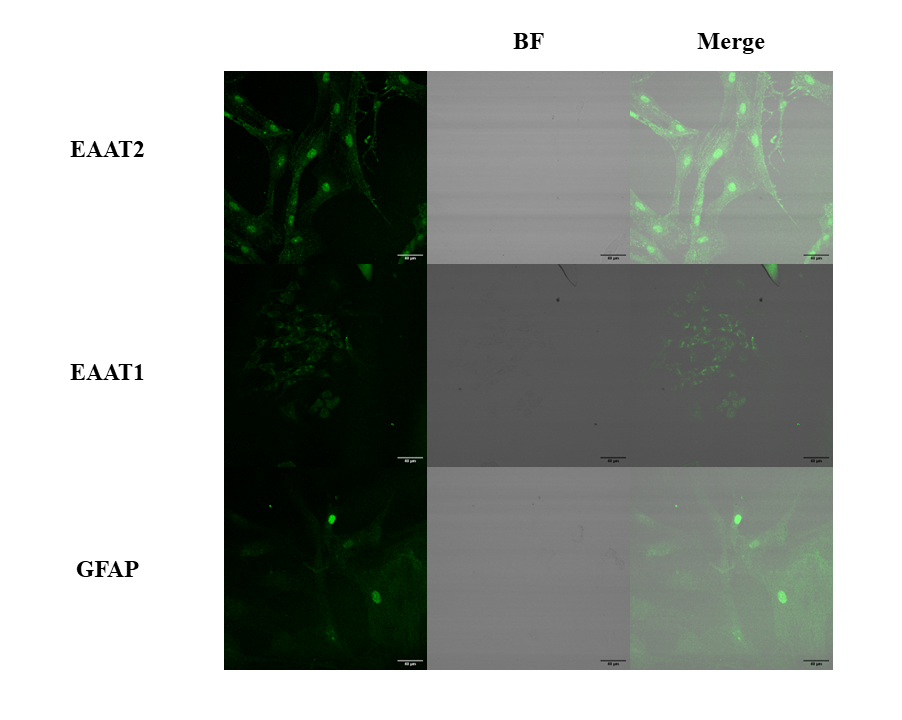


**Figure S11.** Labeling with anti-EAAT1 antibody, anti-EEAT2 antibody, and anti-GFAP antibody to determine cellular composition. Scale bar 40 μm.

According to the immunofluorescent labeling performed, it was determined that 90% of the mixed culture corresponds to astrocytes, and 10% to neurons, microglia, and other glial cells.


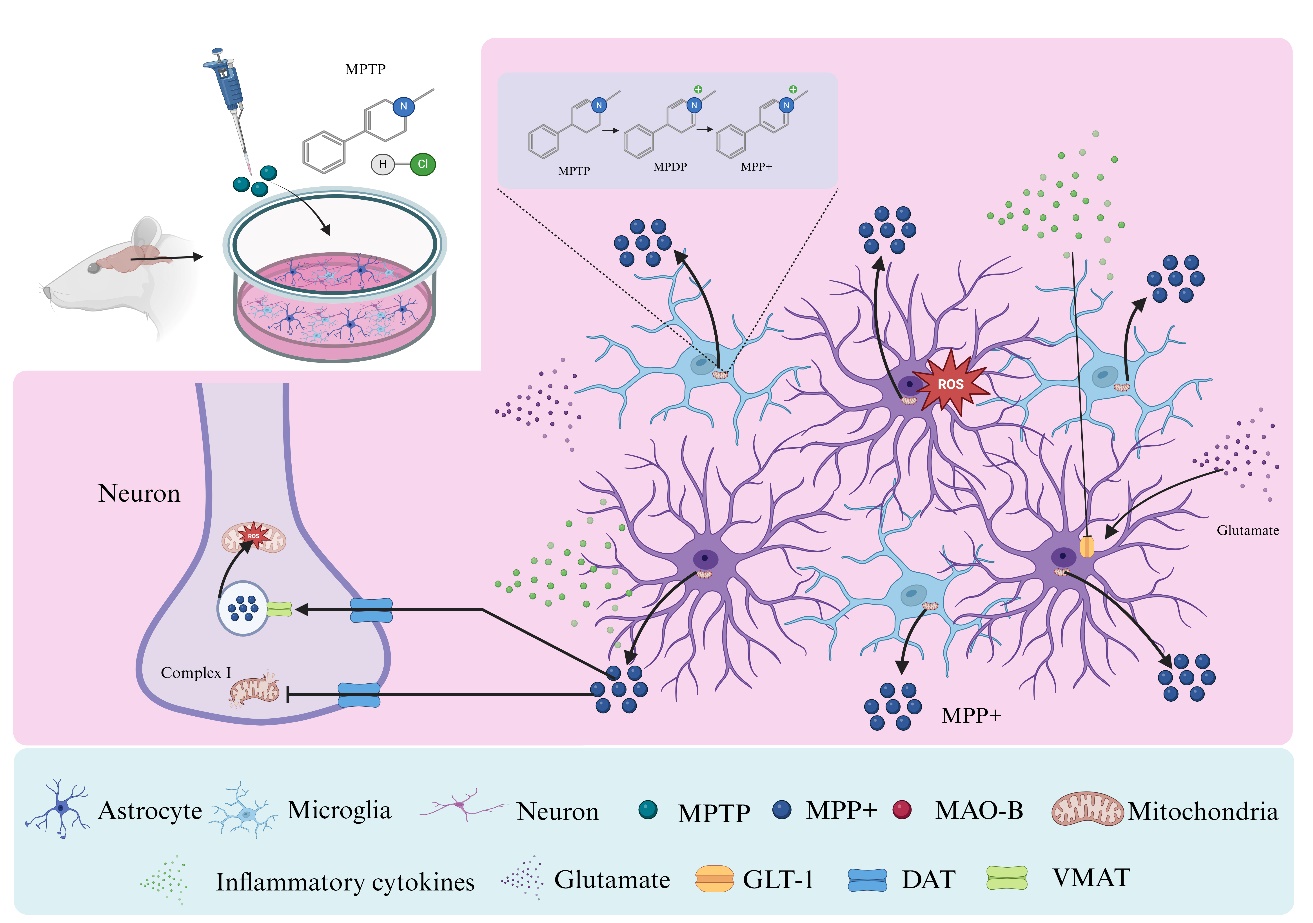


**Figure S12.** Effects of MPTP on cells: MPTP is metabolized to MPP+ by MAO-B. MPP+ enters neurons through the DAT transporter and accumulates in the mitochondria, inhibiting complex I, which leads to oxidative stress. Additionally, MPP+ induces a proinflammatory response and glutamate toxicity.
